# Several areas of overlap between obesity and aging indicate obesity as a biomarker of accelerated aging of human B cell function and antibody responses

**DOI:** 10.1186/s12979-022-00301-z

**Published:** 2022-10-26

**Authors:** Daniela Frasca

**Affiliations:** Department of Microbiology and Immunology and Sylvester Comprehensive Cancer Center, University of Miami Miller School of Medicine, RMSB 3153, 1600 NW 10th Ave, Miami, FL 33136 USA

**Keywords:** Aging, Obesity, Adipose tissue, B cells, Humoral immunity

## Abstract

Aging and obesity are high risk factors for several conditions and diseases. They are both associated with systemic inflammation and they are both ameliorated by a healthy life style, suggesting that they may share cellular and molecular pathways and underlying mechanisms. A close relationship between aging and obesity is also supported by the observation that the aging overweight/obese population is increasing worldwide, and mechanisms involved will be presented here. A focus of our work is to evaluate if obesity may be considered a good biomarker of accelerated aging of human antibody responses. We will summarize our published results showing the effects of obesity in accelerating age defects in the peripheral B cell pool and how these lead to dysfunctional humoral immunity.

## The aging overweight/obese population is increasing

The aging population is increasing worldwide. It is expected that, if this trend is maintained, by 2050 one in four individuals living in Europe and North America will be ≥ 65 years of age, and older adults will outnumber the children [1]. In parallel with the increase in the aging population, there is also an increase in the proportion of older adults that are overweight or obese [2]. This contributes to sarcopenic obesity, a chronic age-related condition due to the interplay between aging, inflammation, unhealthy dietary habits, insulin resistance (IR), sedentary lifestyle, and oxidative stress, leading to a significant decline in muscle mass and a concomitant increase in fat mass [3]. Successful aging and increased lifespan strongly rely on efficient metabolism needed to maximize metabolic efficiency. It is expected, therefore, that with the alarming increase in obesity prevalence life expentancy with be decreased especially in industrialized countries.

Several mechanisms associated with longevity and age-related metabolic dysfunction take place in the adipose tissue (AT), which can be the largest tissue in individuals with obesity. Aging induces changes in body composition and increases the mass of the AT, especially of the visceral AT (VAT), while the subcutaneous AT (SAT) mass decreases [4], as shown by computational tomography scans. VAT and SAT are biologically distinct in terms of secretion of pro-inflammatory mediators, with the VAT being more inflammatory than the SAT. Aging significantly changes the profile of inflammatory mediators produced by the adipocytes, modifying pre-adipocyte number and function and increasing the infiltration of immune cells in the AT [4]. An age-associated increase in the ectopic deposit of triglycerides in several tissues (liver, muscle, heart, pancreas, kidney) [5–9] and in blood vessels [10] has been shown to also occur in elderly individuals that are lean, as identified by body scans and MRI, and has been suggested to be linked to the development and/or progression of age-associated diseases.

The age-associated changes in the AT may be related to a different lifestyle, food choices and eating patterns of older adults that start at the time of retirement, and are in large part responsible for an excessive accumulation of fat in different tissues. Age-induced changes occur in both the lean and the obese AT, leading to changes in energy metabolism and systemic IR. The majority of published studies have clearly demonstrated that obesity, similar to aging, could be a condition of accelerated metabolic dysfunction [11, 12]. This metabolic dysfunction is associated with several medical complications (IR, hyperglycemia, hypertension, and dyslipidemia) also in children and adolescents with obesity, and is responsible for accelerated puberty in girls that has been shown to lead to higher risk of psychological and behavioral problems, as well as to higher probability to develop breast cancer [13, 14]. Studies evaluating immune function in obese children and adolescents, although limited in number, have also indicated significant dysregulation of immune cells [15, 16], characterized by reduced proliferative and differentiative capacity and impaired effector function, leading to reduced anti-tumor and anti-viral immunity [16].

## Shared features between aging and obesity

Aging and obesity are significant risk factors for inflammatory conditions and diseases, such as type-2 diabetes, cancer, psoriasis, atherosclerosis, and inflammatory bowel disease [17, 18], suggesting that they may share cellular and molecular pathways and underlying mechanisms. The pattern of organ-specific deterioration is quite similar during aging and obesity, with obese individuals being considered prematurely aged individuals [19–21]. However, it has also been postulated that the molecular pathways involved in aging and obesity are quite divergent [22].

Although the recently introduced term “adipaging” indicates that aging and obesity share several common features, and obese adult individuals are prematurely aged individuals [23], not so many mechanistic experiments have been performed, even in mice, to link the two processes in a rigorous way. Nevertheless, both aging and obesity have been shown to be associated with systemic inflammation [24–26], oxidative stress [27, 28] and changes in microbiota composition [29, 30]. ROS (Reactive Oxygen Species) is at least one crucial contributor to the immune dysregulation associated with both aging and obesity. ROS in senescent cells as well in cells from the obese AT activates the tumor suppressor gene p53 and leads to telomere damage, IR and cell death [31, 32]. It has been shown that individuals that are overweight or obese have shorter telomeres, markers of cellular senescence, representing another shared feature of aging and obesity [33]. Telomere length has been negatively associated with the atherogenic lipid and lipoprotein pattern of the individuals, further supporting the role of oxidative stress and systemic inflammation, common to both aging and obesity, in telomere attrition. ROS also induces the translocation of the transcription factor NF-kB from the cytoplasm to the nucleus, a signal that leads to increased expression of genes regulating the secretion of pro-inflammatory cytokines, apoptosis, and cell senescence [34, 35]. NF-kB is constitutively activated in immune cells from aged mice and humans following not only oxidative but also metabolic stresses, and a strong link between nutrient sensing and immune signaling has been demonstrated with the convergence of key metabolic and inflammatory signaling pathways [36].

Aging and obesity also share epigenetic changes that include DNA methylation patterns, post-translational modifications of histones, chromatin remodeling and increased production and maturation of RNAs [37–40]. Epigenetic changes occurring during aging and obesity are associated with health issues and several dietary factors have been identified as crucial modifiers of biological age with epigenetic clock models being used to help understanding how nutrition can modulate age-associated diseases and improve health outcomes.

## Interventions to counteract the effects of aging and obesity

Both aging and obesity are ameliorated by a healthy life style [41–43], and the effects of physical activity and healthy diet on reduced risk of diseases [44–47], and the effects of physical activity on improved immunity [48], have been reported. Physical exercise also significantly impacts the methylation of several genes, including those involved in metabolism, fuel usage and muscle growth [49, 50], hematopoiesis [51] and inflammation [52]. Promising results from a small clinical trial have indicated that systemic treatment with a cocktail of the growth hormone dehydroepiandrosterone plus metformin could reverse at least in part the DNA methylation age (DNAmAge) and induce thymus regeneration [53]. Another clinical trial has shown that specific diet and lifestyle interventions may effectively reverse DNAmAge of healthy adult males [54].

Although caloric restriction (CR) has been shown to modulate energy balance and extend maximal lifespan in experimental animal models [55], some reports have indicated that CR may also increase the risk of infections with viruses [56] or parasites [57]. In humans, conversely, the results from the clinical trial CALERIE (Comprehensive Assessment of Long-term Effects of Reducing Intake of Energy) have shown that CR is not decreasing immunological function, and adult participants, who have reduced their calorie intake by 25% over two years, have larger thymuses than control participants who did not restrict calories, and their thymuses have more functional T cells [58]. The study showed that CR induced changes in gene activity in both non immune and immune cells in the AT. One of these genes, Pla2g7, regulates inflammation, and CR significantly inhibited its activity. B cell responses were not evaluated in this study but we can hypothesize that they could also be increased.

## Common effects of aging and obesity on B cell function and humoral immunity

Antibody responses to influenza vaccination decrease with age, with the quantity and the quality [59–63] as well as duration of circulating antibodies being decreased [64]. Influenza vaccine-specific antibodies do not persist year-round in older adults [65]. For this reason, vaccinated elderly individuals can still be infected and with severe secondary complications such as hospitalization, catastrophic disability, exacerbation of underlying medical conditions and death [66–68], due to their compromised immunity. In addition to age, preexisting immunity, genetic polymorphisms, and the presence of chronic underlying conditions may compromise the influenza vaccine response in this vulnerable population [69–71].

In order to evaluate if obesity may accelerate age defects in humoral immunity, we measured the serum response to the influenza vaccine in young and elderly healthy individuals, both lean and obese, by hemagglutination inhibition assay, which is the best correlate for vaccine protection, and found that obesity is significantly associated with decreased in vivo responses to the vaccine in both age groups [72]. To our knowledge, we have been the first and only ones so far to show the effects of obesity on the in vivo influenza vaccine response in both young and elderly individuals. Our results have confirmed and extended those from other studies showing decreased influenza vaccine-specific antibody responses in serum samples of adult obese individuals as compare to those from lean controls, with BMI being positively correlated with the decline in serum antibodies [73, 74]. What we found particularly interesting in our study was that the vaccine-specific response of young obese individuals was not different from that of elderly lean individuals, suggesting that obesity, by inducing age defects in the humoral response to the influenza vaccine, may be a biomarker of accelerated aging at least for antibody responses. Obesity, similar to aging, induced defects in class switch recombination (CSR) and somatic hypermutation (SHM), two processes necessary for the generation of class switched high affinity secondary antibodies [75]. Defects in CSR and SHM are due to reduced expression of activation-induced cytidine deaminase (AID), the enzyme of CSR and SHM, and E47, encoded by the E2A gene, a key transcription factor regulating AID [76]. Both AID and E47 are decreased in B cells isolated from the blood of obese young and elderly individuals as compared to lean controls [72]. Again, and very important, the response of elderly lean individuals was not different from that of young obese individuals. In further support of our hypothesis, we have also found comparable amounts of IgG antibodies with autoimmune specificity in serum samples of young obese and elderly lean individuals [77].

When we evaluated the B cell pool of young and elderly lean participants, we found a significant expansion of a subset of B cells that is the most pro-inflammatory, called Double Negative (DN) B cells, as previously reported by us [78, 79] and by others [80]. These cells have been identified as CD19+CD27-IgD- and have been shown to be increased in the blood of patients with inflammatory conditions including autoimmune [81–83] and chronic infectious diseases [84–86]. We found their frequencies in blood negatively associated with the influenza vaccine response [72, 78], as expected, due to the fact that DN B cells secrete large amounts of pro-inflammatory cytokines making them refractory to further stimulation.

In a subsequent study, we found that the blood of obese young individuals is also significantly enriched in DN B cells, with DN B cell frequencies being similar to those observed in the blood of elderly donors, confirming that obesity and aging are inducing similar changes in the peripheral B cell pool [87, 88]. DN frequencies were even higher in the AT of obese donors and in some individuals frequencies reached 80% of total B cells [88]. While DN B cells do not proliferate and do not make antibodies to “new” antigens, they secrete antibodies that are autoimmune and specific for intracellular proteins, ubiquitously expressed in different tissues of the organism, released following hypoxia and cell death occurring at high rates in the calorie-stressed AT. DN B cells have the membrane phenotype of autoimmune B cells (CD95+CD21-CD11c+) and spontaneously express T-bet, the transcription factor for the secretion of autoimmune antibodies, as we have shown in our human [88] and mouse [89] studies. In support of the above results, previously published work has also clearly shown that the plasma of obese individuals that are insulin resistant contains autoantibodies specific for these intracellular proteins, suggesting the chronic release of “self” antigens by the AT under obesity conditions.

Recently, to define at least one possible mechanism through which obesity, similar to aging, induces the secretion of IgG antibodies with autoimmune specificity, we used an in vitro model in which B cells from young and elderly lean donors have been stimulated with the Fatty Acid (FA) palmitate. Palmitate is the salt of palmitic acid, the most common saturated FA in the human body, accounting for > 60% of total saturated FAs in the body and > 30% of total FAs in blood [90, 91]. The rationale to perform these experiments was that there is a chronic increase in blood levels of the FA palmitate, due to increased spontaneous lipolysis occurring during aging and obesity, and this may induce autoimmune (pathogenic) B cells. Our results have indeed shown that the in vitro incubation of B cells from lean young and elderly donors with the FA palmitate induces mRNA expression of T-bet, as well as secretion of autoimmune IgG antibodies, with B cells from young lean individuals looking similar to B cells from elderly lean donors, confirming our initial hypothesis and also showing the critical role of the FA palmitate in inducing human B cell immunosenescence [77].

The effects of aging and obesity on human B cells are summarized in Table 1. To our knowledge, divergent features between these two conditions on human B cells have not been reported so far.

**Table 1:** Effects of aging and obesity on human B cells

	Aging	References	Obesity	References
Influenza vaccine response^a^	↓	59,60,61,62,63,71,72	↓	72,73,74
In vitro class switch				
*E47*^b^	↓	72	↓	72
*AID*^c^	↓	72	↓	72
In vitro pro-inflammatory cytokine secretion				
*IL-6*	↑	72	↑	72
Intrinsic inflammation				
*icTNF-α*	↑	72	↑	72
*RNA expression of SASP markers*^d^	↑	72	↑	72
Frequencies of DN B cells in blood	↑	78,79,80,88	↑	72,87,88

^a^Measured by hemagglutination inhibition assay

^b^Transcription factor of class switch

^c^Activation-induced cytidine deaminase, the enzyme of class switch recombination and somatic hypermutation

^d^*SASP* Senescence-associated secretory phenotype

## Conclusions

The mechanisms by which aging and obesity decrease protective humoral responses and increase autoimmunity are in large part overlapping, although our knowledge of the cellular and molecular pathways involved is still only partial. Obesity accelerates inflammaging and immunosenescence as well as metabolic, physiological, and functional changes in immune cells that are associated with dysfunctional immunity. Although in the literature many reports have clearly outlined the relationship between excessive fat accumulation and the aging process, future studies are still needed to provide additional mechanisms and further support the notion that obesity is a valid biomarker of accelerated immune aging. Given the increased prevalence of obesity in Western countries, these studies will be highly relevant for public health and will allow the identification of targets for prevention and/or therapy.

## Data Availability

Not applicable.
